# Effect of date molasses on levetiracetam pharmacokinetics in healthy rats

**DOI:** 10.1038/s41598-023-28074-5

**Published:** 2023-01-14

**Authors:** Wael Abu Dayyih, Raghad Layth, Mohammad Hailat, Bayan Alkhawaja, Lina Al Tamimi, Zainab Zakaraya, Aseel Aburumman, Nisreen Al Dmour, Mohamed J. Saadh, Hisham Al-Matubsi, Saed M. Aldalaen

**Affiliations:** 1 Faculty of Pharmacy, Mutah University, Al-Karak, Jordan; 2 Faculty of Pharmacy and Medical Sciences, University of Petra, Amman, Jordan; 3 Faculty of Pharmacy, Al-Zaytoonah University of Jordan, Amman, Jordan; 4 Faculty of Pharmacy, Zarqa University, Zarqa, Jordan; 5 Faculty of Pharmacy, Al-Ahliyya Amman University, Amman, Jordan; 6 Pharmacological and Diagnostic Research Centre, Faculty of Pharmacy, Al-Ahliyya Amman University, Amman, Jordan; 7 Faculty of Agriculture, Mutah University, Al-Karak, Jordan; 8 Faculty of Pharmacy, Middle East University, Amman, 11831 Jordan; 9 Department of Pharmacology, Faculty of Medicine, Mutah University, Al-Karak, Jordan

**Keywords:** Pharmacology, Neurological disorders, Drug regulation, Drug safety, Pharmaceutics, Pharmacology

## Abstract

Twelve healthy eight-week-old male Wistar rats weighing 200 g were used. Rats were chosen randomly, and their tails were identified and separated into cages/groups. The first group received an oral dose of 11.5 mg of levetiracetam in 5 mL of water, and the second group was given date syrup (250 g mixed with 250 mL water) for seven days, then 11.5 mg LEV in 5 mL water on day 7. One week of preadministered date molasses significantly decreased levetiracetam pharmacokinetic parameters in rats, such as C_max_ (72 vs. 14 ng/mL, *p* = 0.01), T_max_ (1.78 vs. 0.44 h, *p* < 0.001), and AUC (880 vs. 258 ng.h/mL, *p* < 0.001). This decrease in plasma levetiracetam levels caused by date molasses could be attributed to decreased levetiracetam absorption. On the other hand, the current study discovered that rats given date molasses for a week had a reduced rate and extent of absorption. As a result, date molasses might increase the risk of epileptic seizures in oral LEV-treated ones.

## Introduction

Epilepsy is a debilitating and widespread neurological disorder defined by aberrant electrical activity in the brain, which results in seizures or odd behavior, sensations, and even loss of consciousness^1^. Epilepsy affects an estimated 50 million people worldwide, with 75 percent of those living in resource-poor countries having limited or no medical care or treatment access. Around 50 per 100,000 people in developed countries are estimated to have epilepsy yearly. In countries with limited resources, the annual incidence of epilepsy ranges from 100 to 190 per 100,000 people^2^. Epilepsy patients often take the anticonvulsant drug levetiracetam (LEV). Focal-onset seizures (seizures affecting only a portion of the brain) in adults, children, and infants aged one month and older can be controlled with LEV, either independently or in combination with other medications. It calms the brain's overactive excitability^3,4^. In conjunction with epileptic treatment, low-sugar diets like the ketogenic diet (KD) have improved epileptic seizure symptoms^5,6^. KD is usually recommended if a child's seizures have not improved after taking several medications. People following KD follow a low-calorie, low-protein, and low-carbohydrate diet to lose weight. If you follow the "long-chain triglyceride diet," you will get about 3 to 4 g of fat for every 1 g of carbs and protein^7^. According to recent studies, refractory epilepsy in children and adults can be successfully treated with KD^8–11^.

Additionally, "date molasses" can refer to several different products. It has a dark ruby red hue and a thick, sticky sweetness to the liquid. By far the most common date-derived product is date syrup, which can be made in three ways: (i) as an accidental byproduct of storing bagged, humid dates (especially in the Gulf area), (ii) at the home or village level by extraction and boiling down of the juice, and on a semi- and full-industrial scale. It costs about 9.5 cents per kilogram of date molasses^12^. In Middle Eastern countries, it is a necessity. It is loaded with essential vitamins, minerals, and polysaccharides. It also contains a small number of proteins. In addition to the previously mentioned antioxidants and vitamins/minerals, these also contain iron, calcium, magnesium, vitamin B6, and selenium, all of which are essential nutrients for human health^13^.

As a food supplement commonly found in Mediterranean households, we hypothesized that the polysaccharides in date molasses might interact with LEV and reduce its pharmacokinetic parameters. However, date molasses has never been studied concerning the pharmacokinetics of LEV in vivo, and there are no studies on date molasses interaction. This is something we are going to investigate further. As a result, the study aims to determine the effects of date molasses oral administration on rat LEV pharmacokinetics.

## Materials and methods

### Animal and in vivo experimental design

The study used twelve healthy eight-week-old male Wistar rats weighing 200 g, which had been chosen by utilizing the 3 R’s rule^14^. Before beginning the study, rats were randomly chosen, had their tails identified, and placed in separate cages/groups. A week before the study began, all rats were acclimatized, fed regular food, and water was ad libitum.

Rats were divided into two groups. Group one was administered an oral dose of 11.5 mg of LEV in 5 mL of water, and group two was kept only drinking for seven days' date syrup (dates molasses; 250 g mixed with 250 mL water) and then, on day seven an oral dose of 11.5 mg of LEV in 5 mL of water was administered.

### Chemicals

LEV was supplied from Hikma, Jordan, with a potency of 99.8%, whereas the following reagents and chemicals were obtained from Merck (Darmstadt, Germany): orthophosphoric acid (AR grade 85%), acetonitrile (HPLC grade), and Milli-Q purified water.

### High-performance liquid chromatography (HPLC)

The HPLC system used was Thermo Finnigan Surveyor UV–VIS with a detector, LC Pump (SRVYR-LPUMP), and autosampler (SRVYR-AS). The column used was ACE C18-AR (250 mm*4.6 mm) 5 µm, an average particle size.

### The mobile phase, internal standard, and standard solutions

The buffer solution was prepared by adding 3.4 g KH_2_PO_4_ to 1000 mL of HPLC- grade water and pH adjusted to 5.2 with orthophosphoric acid. The mobile phase was prepared by mixing 830 mL of the buffer solution with 170 mL of acetonitrile and orthophosphoric acid. The mixture was then filtered using a 0.45 μm membrane filter and degassed by sonication. The internal standard (IS) was prepared by weighing 3 mg of pure metformin hydrochloride and dissolving it in 100 mL of freshly prepared acetonitrile. Then 1 mL of it was diluted with 200 mL of acetonitrile.

### Preparation of stock solutions and working solutions

Stock solutions of LEV were prepared by weighing and transferring 20 mg of each active ingredient into a 100 mL volumetric flask and diluted up to the mark with ACN.

Preparation of the working standard solutions:

Step Dilution 1:2.5 mL of LEV solution was pipetted into a 20 mL volumetric flask.

Concentration, then diluted to seven concentrations 10, 25, 50, 75, 100, 500, 1000 ng.

For the sample solution for LEV preparation, a volume of plasma was pipetted in Eppendorf and diluted with IS, then vortex for 1 min, and then centrifuged for 15 min. The supernatant was injected into the HPLC.

### Wavelength selection

UV–VIS scan applied for the solution of LEV was within a range of 200 – 400 nm. Maximum absorbance of 205 nm was obtained.

### Method development

**Figure 1:**
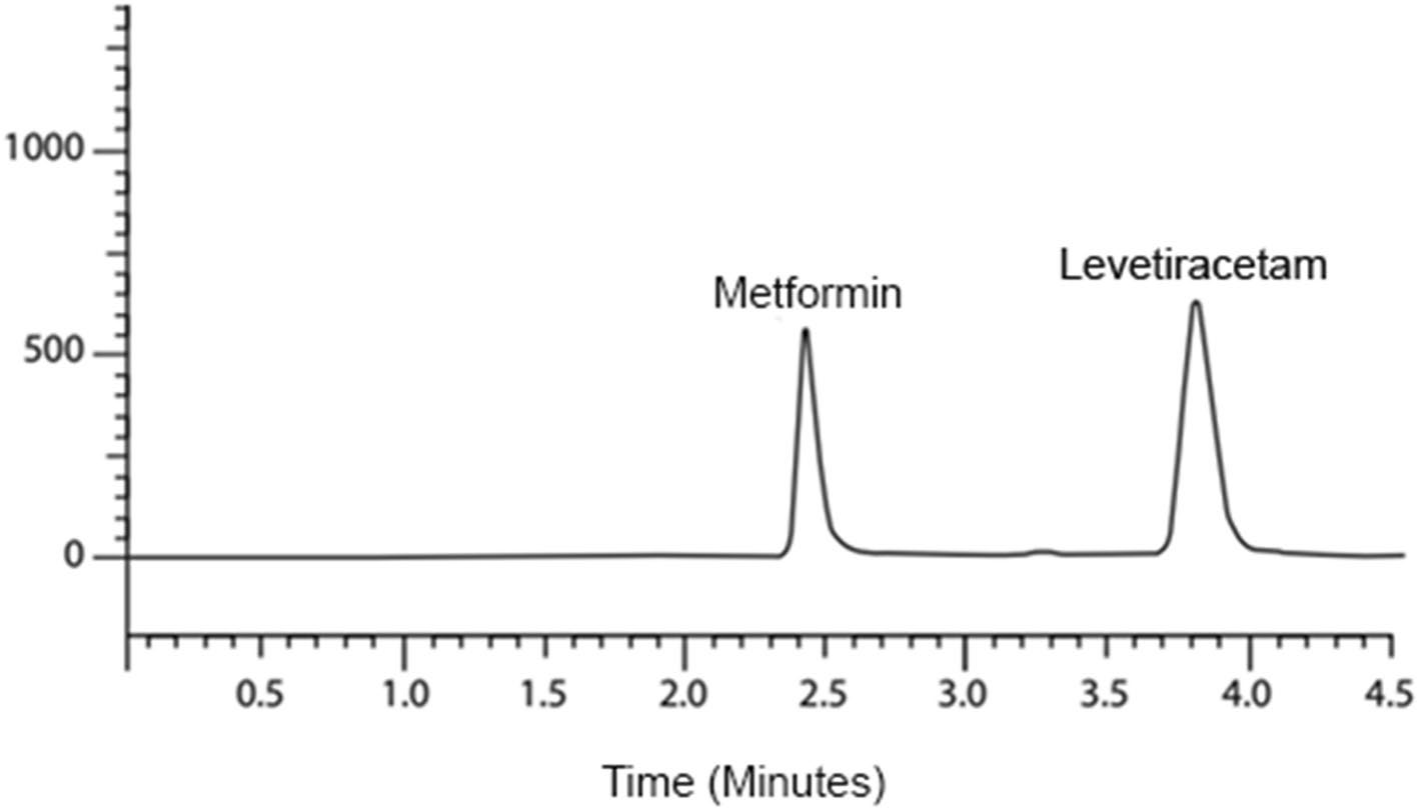
A chromatogram of a sample containing a mixture of LEV and metformin at optimum conditions.

The method of development of the study was to best chromatographic conditions for assaying LEV using metformin as an IS, such as having a short retention time with good resolution and symmetrical peaks (Fig. 1). Different factors such as pH, ion pair, the composition of mobile phase, and column were evaluated.

After three trials for chromatographic conditions, in which two were rejected, we found the best resolution at pH 5.2, wavelength: 210 nm, for 10 µl injection volume, flow rate 1 mL / min, and oven temperature 25 °C.

For pharmacokinetic analysis, blood samples were withdrawn from the rats before and immediately after treatment and at 0.33, 0.66, 1, 2, 4, 8, 24, 36, 72, and 96 h.

### Pharmacokinetic analysis

The term "relative bioavailability" refers to the comparison of two formulations (or two methods of administration of the same formulation) without the use of an intravenous injection^15^. In addition if the two administered routes give the same percent, i.e., 100%, they are equivalent to each other. The maximum plasma LEV concentration (C_max_) and time to reach the maximum concentration (T_max_) were calculated by averaging the highest plasma concentration and its corresponding time of LEV in each rat. Besides, the area under the curve under the plasma concentration–time profile from time zero to time t (AUC_t_), zero to infinity (AUC_0⟶∞_), and elimination half-life (t_1/2_) were calculated based on non-compartmental pharmacokinetic calculations.

Furthermore, AUC_0⟶∞_ was calculated by adding AUC_t_ to the value of dividing the last measurable concentration at time T over the elimination rate constant. The t_1/2_ was calculated from the slope of the semi-logarithmic of the last plasma concentration point vs. time.

### Data analysis

The PK parameters; C_max_, T_max_, AUC_t_ (AUC_0⟶∞_), and t_1/2_ were presented as mean (± SD). One-way analysis of variance was used to calculate the significant differences of each parameter between the groups, followed by Tukey as a post hoc test to determine the differences between each group.

### Ethics approval

This study was carried out in accordance with ARRIVE guidelines and was approved by the Scientific Research and Ethics Committee [SREC]–Faculty of Pharmacy/ Mutah University, Al-Karak 61,710, Jordan. SREC 3–2022/11 / Feb. 3, 2022 is the registration code that was obtained by adhering to the Declaration of Helsinki's ethical standards for human research and the International Conference on Harmonization's Good Clinical Practice Guidelines, and the NMPA's Guideline for Good Clinical Practice.

## Results

### Pharmacokinetics of LEV in rats with and without taking of date molasses

Table 1 shows that rats given date molasses for a week had a reduced rate and extent of absorption. Compared with the control group, the oral pharmacokinetics of LEV, when combined with date molasses, were altered.

**Table 1:** The pharmacokinetic parameters of LEV after oral administration to rats with and without date molasses (mean SD).

Route of administration	AUC	T_max_	C_max_
	Mean ± SD	Mean ± SD	Mean ± SD
LEV oral	880 ± 306	1.78 ± 0.55	72.1 ± 50.7
LEV oral + dates	258 ± 38**	0.44 ± 0.17**	14.1 ± 5.9*

*p < 0.05 less than LEV oral.

**p < 0.001 less than LEV oral.

## Discussion

Plasma concentrations represent a time curve from medication administration to peak effect and final elimination. The time to peak concentration is essential in practice because it varies across people and may be adjusted by different routes of administration (e.g., oral, IV, and topical) or through changes to the drug delivery mechanism. T_max_ is often tied to the duration of a drug's half-life (t_1/2_). Drugs have a short half-life peak and disappear fast, necessitating more frequent doses to keep the medicine within its clinically effective therapeutic range^16^. The area under the curve (AUC) comparing blood concentration vs. time is significantly linked with maximum concentration (C_max_). However, if the drug's elimination is linear, the AUC will be proportionate to the dosage. When the medication clearance is increased, the AUC decreases. The medication spends less time in the systemic circulation, and its plasma drug concentration decreases more quickly with increased clearance. Therefore, the total amount of medication absorbed by the body and the area under the concentration–time curve are reduced under these conditions^17^.

The pharmacokinetics of LEV after oral administration of date molasses resulted in a statistically significant decrease of T_max_ (from 1.78 ± 0.55 to 0.44 ± 0.17 h ± SD, p < 0.001 less than LEV oral), AUC (from 880 ± 306 to 258 ± 38 ng/mL ± SD, p < 0.001 less than LEV oral), and C_max_ (from 72.1 ± 50.7 to 14.1 ± 5.9 ng/mL ± SD, p < 0.05 less than LEV oral).

**Figure 2:**
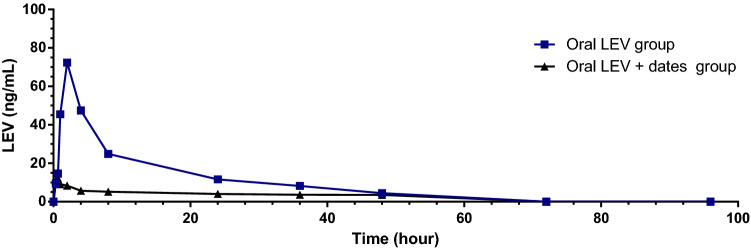
Pharmacokinetics profile of LEV administered orally or orally together with date molasses (*n* = 6).

A one-compartment model with first-order absorption and elimination adequately represented LEV pharmacokinetics^18^. Administering a combination of drugs and or food may alter their pharmacokinetics^19,20^. This study investigated the combination of date fruit molasses on LEV Pharmacokinetics in healthy rats. Where one week of pre-administrated date molasses significantly decreased LEV pharmacokinetic parameters in rats; C_max_ (72 vs. 14 ng/mL, *p* = 0.01), T_max_ (1.78 vs. 0.44 h, *p* < 0.001), and AUC (880 vs. 258 ng.h/mL, *p* < 0.001) (Table 1, and Fig. 2). This decrease in plasma LEV levels caused by date molasses co-administration could be attributed to an increased elimination rate of LEV associated with the faster absorption rate (i.e., T_max_ decreased from 1.78 ± 0.55 to 0.44 ± 0.17 h). However, a previous study found that food slows the rate of LEV absorption but not the extent of absorption^21^. The latter conclusion was reached because LEV AUC did not differ without and with food. On the contrary, the current study found that rats given date molasses for a week had a reduced rate and extent of absorption.

Furthermore, it has been demonstrated that combining a low-sugar diet, such as the ketogenic diet, with epileptic treatment reduces epileptic seizures^5,6^. Polysaccharides in date molasses could theoretically increase the risk of epileptic seizures. As a result, more research is needed to verify this. When taking LEV to control epileptic seizures, doctors and patients should be advised to limit their sugar or date molasses intake. Our significant findings also necessitate further human research to examine the potential consequences on patients where the drug's activity may decrease.

Approximately 34% of LEV dosage is metabolized, with the remaining 66% eliminated in urine unmetabolized; however, the metabolism is not hepatic but instead happens mainly in the blood through hydrolysis^21^. As a result, date molasses is proposed to increase LEV clearance by inducing clearance through urine elimination.

## Data Availability

All data generated or analyzed during this study are included in this published article.
